# Expert Opinion of Empagliflozin Positioning in the Current Type 2 Diabetes Mellitus (T2DM) Landscape in Indian Patients

**DOI:** 10.7759/cureus.99023

**Published:** 2025-12-12

**Authors:** Bipin Sethi, Ashwani Mehta, Arundhati Dasgupta, Manoj Chawla, Nilakshi Deka, Navneet Agarwal, Chinmoy Mazumder, Ipshita Ghosh, Vishal Gala, Sameer Muchhala, Akanksha Sonkar

**Affiliations:** 1 Endocrinology, CARE Hospitals Outpatient Centre, Hyderabad, IND; 2 Cardiology, Sir Ganga Ram Hospital, New Delhi, IND; 3 Endocrinology, Rudraksh Superspeciality Care, Siliguri, IND; 4 Endocrinology and Diabetes, Lina Diabetes Care Centre, Mumbai, IND; 5 Endocrinology, Diabetes and Metabolism, Apollo Hospitals, Guwahati, IND; 6 Medicine, Diabetes, Obesity &amp; Thyroid Centre (DOTC) Gwalior, Gwalior, IND; 7 Internal Medicine, Vikrant University, Gwalior, IND; 8 Endocrinology and Diabetes, Tulip Mediworld - Superspeciality Hospital, Guwahati, IND; 9 Endocrinology, Vivekananda Institute of Medical Sciences, Kolkata, IND; 10 Medical Affairs, Zydus Healthcare Limited, Mumbai, IND; 11 Medical Affairs, Zydus Lifesciences Limited, Mumbai, IND

**Keywords:** empagliflozin, glp-1, masld, sglt2 inhibitors, t2dm

## Abstract

Background

Type 2 diabetes mellitus (T2DM) is a complex metabolic disorder associated with significant macrovascular and microvascular complications. Empagliflozin, a sodium-glucose cotransporter 2 (SGLT2) inhibitor, has demonstrated benefits beyond glycemic control, including cardiovascular and renal protection. As a result, it is increasingly prescribed for patients with T2DM who are at an elevated risk of adverse cardiorenal outcomes, specifically those with established atherosclerotic cardiovascular disease (ASCVD), heart failure (HF), or chronic kidney disease (CKD).

Objective

This study aimed to evaluate clinicians' perspectives on the role of empagliflozin in the management of T2DM in India, particularly among patients with cardiovascular, renal, and metabolic comorbidities.

Methods

A structured new age for a virtual advisory board was conducted with overall 625 experts, including endocrinologists, diabetologists, and cardiologists from diverse regions of India. The 16-item questionnaire used in this advisory board was developed by an expert panel based on published evidence and clinical experience; however, it was not formally validated or pilot-tested prior to administration. As such, responses may reflect expert opinion and clinical judgment rather than performance on a standardized, validated instrument.

Results

Strong consensus was observed across key domains: 95% agreed that empagliflozin should be a foundational therapy for T2DM patients with ASCVD, while 93% supported its use for HF prevention across all ejection fractions, and 93% endorsed its role in slowing the progression of diabetic kidney disease. Early initiation in high cardiorenal risk patients, even before metformin, was favored by 90%. Additionally, 92% supported its use in non-diabetic HF and CKD patients. Notably, 83% of clinicians perceived empagliflozin to have a superior clinical profile compared with other SGLT2 inhibitors; however, this reflects expert perception rather than direct comparative evidence, and many benefits are recognized as class effects across the SGLT2 inhibitor group. A favorable safety profile was affirmed by 86% of experts, while additional benefits such as metabolic dysfunction-associated steatotic liver disease (MASLD) improvement (86%), quality-of-life enhancement (90%), and uric acid reduction (84%) were also acknowledged based on clinical experience and emerging evidence.

Conclusion

This expert consensus highlights widespread clinical support for empagliflozin as a cornerstone therapy in T2DM, especially for patients with cardiovascular, renal, or metabolic risk. Its multidimensional benefits support early initiation and broad application in Indian clinical practice.

## Introduction

Diabetes mellitus refers to a group of metabolic conditions involving disrupted carbohydrate metabolism, where glucose is insufficiently used for energy and excessively generated through abnormal gluconeogenesis and glycogenolysis, leading to elevated blood glucose levels (hyperglycemia) [1]. It is a progressive disorder with severe complications, increasing healthcare costs owing to its chronic nature. Uncontrolled diabetes elevates vascular disease risk, with macrovascular (cardiovascular, cerebrovascular, and peripheral arteries) and microvascular (retinopathy, nephropathy, and neuropathy) complications [2]. Type 2 diabetes mellitus (T2DM) is a heterogeneous, complex disorder involving multiple pathophysiological mechanisms affecting the pancreas and metabolic organs, complicating treatment [3].

In 2019, the World Health Organization (WHO) reported that noncommunicable diseases (NCDs) caused 74% of global deaths, with diabetes responsible for 1.6 million, ranking ninth worldwide. According to the International Diabetes Federation (IDF) Diabetes Atlas, 11th Edition, an estimated 589 million adults aged 20-79 (11.1%) worldwide have diabetes, and this number is projected to rise by 45% to 852.5 million by 2050. India is among the major contributors to the global diabetes burden, with a rapidly rising cardiometabolic risk profile [4]. In India, T2DM showed a fourfold rise in the disability-adjusted life year (DALY) rate, ranking 13th in 2016 from 35th in 1990 [5]. Diabetes prevalence was higher in urban (11.2%) than rural (5.2%) areas, with prediabetes exceeding diabetes in most states. Asian Indians also progress faster from prediabetes to diabetes compared to other ethnic groups [6,7]. Given this high burden and the strong clustering of atherosclerotic cardiovascular disease (ASCVD), heart failure (HF), and chronic kidney disease (CKD) in Indian patients, therapies offering benefits beyond glucose lowering are increasingly relevant.

Recent American Diabetes Association/European Association for the Study of Diabetes (ADA/EASD) consensus reports emphasize an individualized approach to T2DM management, recommending glucose-lowering therapies based on comorbidities rather than hemoglobin A1c (HbA1c) alone. In particular, sodium-glucose cotransporter 2 (SGLT2) inhibitors and glucagon-like peptide-1 (GLP-1) receptor agonists with proven cardiovascular and renal benefits are prioritized for patients with established ASCVD, HF, or CKD independent of baseline HbA1c or metformin use. Other available therapeutic classes include dipeptidyl peptidase 4 (DPP-4) inhibitors, sulfonylureas, thiazolidinediones, and basal insulin, which may be incorporated based on patient-specific needs and clinical context [8,9]. SGLT2 inhibitors reduce renal glucose reabsorption by blocking SGLT2 in the proximal tubule (S1 and S2), thereby promoting glycosuria and improving glycemic control [10].

Empagliflozin, an oral SGLT2 inhibitor, was approved in 2014 by the European Commission and the US Food and Drug Administration (FDA) for T2DM management [11]. SGLT2 inhibitors were subsequently approved for CKD in 2021 and received a Class I recommendation for treating HF with reduced ejection fraction (HFrEF) per the American College of Cardiology/American Heart Association/Heart Failure Society of America (ACC/AHA/HFSA) guidelines in 2022 [12,13]. Among currently available agents, empagliflozin demonstrates high selectivity for SGLT2 over SGLT1 (2500-fold) [11,14]. Beyond glycemic control, its natriuretic effects, hemodynamic benefits, and modulation of the renin-angiotensin-aldosterone system contribute to its cardiovascular and renal protective properties [15,16]. Clinical studies have shown significant improvements in glycemic control, body weight, and systolic blood pressure with empagliflozin as monotherapy or combination therapy [17-23,15], along with reductions in HF-related hospitalization and mortality [24]. In Indian populations, empagliflozin has been shown to be safe and effective for glycemic control, weight reduction, and blood pressure improvement [25].

Given the expanding cardiorenal indications of SGLT2 inhibitors and the unmet need for early, organ-protective therapies in India, it is important to understand how clinicians perceive the role of empagliflozin in real-world practice. This advisory board aimed to capture expert perspectives on its positioning across cardiometabolic conditions in the Indian setting.

## Materials and methods

Structured virtual advisory board meetings were conducted between March 6, 2025, and May 23, 2025, to obtain expert clinical insights on the use of empagliflozin in the management of T2DM, with a particular focus on the Indian population. The meeting was held on a secure digital platform to enable broad participation and foster interactive discussions. The format included pre-meeting survey responses, live presentations, and moderated discussions aimed at generating evidence-based recommendations.

The advisory board comprised a multidisciplinary panel of 572 experts, including endocrinologists, diabetologists, cardiologists, and specialists with relevant experience in managing T2DM and related comorbidities. Experts were eligible to participate if they had a minimum of 5-10 years of independent clinical practice and held at least an MD (Medicine) qualification. Only clinicians actively treating adult T2DM patients were included. Exclusion criteria comprised pediatric practitioners, non-clinical roles, non-practicing clinicians, and any specialties unrelated to adult metabolic or endocrine care. No requirement for membership in a specific national society was applied, and no professional organization formally endorsed the advisory process [26]. The selection aimed to ensure geographical diversity and representation from both public and private healthcare sectors across India.

While traditional sample size calculations (as used in clinical trials or population surveys) do not apply to qualitative expert advisory board studies, the selection of 572 experts was driven by the need for comprehensive representation. This sample size was specifically chosen to reflect the diversity of clinical experience and geographic distribution across India. The goal was to ensure that a wide range of viewpoints from multiple specialties (e.g., endocrinology, cardiology, nephrology) was represented to produce well-rounded and actionable insights.

The 16-item questionnaire was developed specifically for this advisory board following a thorough review of the current literature, clinical guidelines, and expert inputs [1,26-28]. It encompassed several key thematic areas, including the cardiovascular benefits of therapy, renal protection and the progression of diabetic kidney disease (DKD), metabolic effects, and the overall safety profile with an emphasis on risk-benefit considerations. Additional domains explored other potential indications for use, comparisons with alternative SGLT2 inhibitors, and the impact of treatment on patients' overall quality of life (QoL).

Responses were collected using a three-point Likert scale (Strongly Agree, Agree, Disagree) in two phases: a pre-meeting survey, in which experts completed the questionnaire prior to the advisory board, and live polling during the discussion, where each question was revisited in real time. A simplified three-point Likert scale was intentionally selected to facilitate clear and rapid consensus formation in a large national advisory board and to reduce variability associated with intermediate response categories. This format is commonly used in expert consensus exercises where the primary objective is to determine directional agreement rather than measure nuanced gradations of opinion. In the results, "NA" indicated that a participant did not answer a given question or felt the item did not apply to their clinical practice. Descriptive statistics were used to analyze the responses. Points of clinical divergence were explored during the meeting to identify key barriers and considerations influencing therapeutic decision-making. Qualitative insights from these discussions were documented to contextualize the consensus outcomes and support the development of expert recommendations.

This activity was a non-interventional, virtual advisory board conducted with licensed clinicians and did not involve human or animal subjects, identifiable patient data, or any experimental intervention. As such, it was exempt from institutional review board (IRB) or independent ethics committee (IEC) review and approval.

## Results

A total of 572 clinicians participated in the consensus survey assessing the perceived clinical utility of empagliflozin in the management of T2DM, particularly in patients with cardiovascular and renal comorbidities. Responses were analyzed to determine the level of agreement across 16 targeted clinical questions. A comprehensive review of clinician responses in Table 1 reveals a strong, multidimensional trend of agreement regarding the use of empagliflozin across various high-risk clinical scenarios. Overall, 95% of clinicians marked "Agree" or "Strongly Agree" with the statement that empagliflozin may be a preferred option for T2DM patients with established ASCVD. 

**Table 1:** Results of advisory board outcomes T2DM: type 2 diabetes mellitus; ASCVD: atherosclerotic cardiovascular disease; HFrEF: heart failure with reduced ejection fraction; HFpEF: heart failure with preserved ejection fraction; DKD: diabetic kidney disease; CKD: chronic kidney disease; SGLT2: sodium-glucose cotransporter 2; AKI: acute kidney injury; DKA: diabetic ketoacidosis; PAD: peripheral artery disease; MASLD: metabolic dysfunction-associated steatotic liver disease; QoL: quality of life

Q. no.	Full question	Agree		Strongly Agree		Disagree		NA		Total
		n	%	n	%	n	%	n	%	
1	Do you agree that empagliflozin should be a foundational therapy for T2DM patients with ASCVD based on current evidence?	97	17%	449	78%	0	0%	26	5%	572
2	In your clinical practice, do you prefer empagliflozin for heart failure prevention in T2DM, regardless of ejection fraction (HFrEF and HFpEF)?	170	30%	362	63%	9	2%	31	5%	572
3	Based on available evidence, do you support empagliflozin as the preferred agent for slowing DKD in T2DM?	192	34%	340	59%	6	1%	34	6%	572
4	Do you agree that empagliflozin should be considered early in T2DM management, before metformin, in patients with high cardiovascular or renal risk?	204	36%	309	54%	25	4%	34	6%	572
5	Does empagliflozin's blood pressure reduction in T2DM significantly impact clinical decisions for hypertensive patients?	244	43%	217	38%	76	13%	35	6%	572
6	In real-world clinical settings, does empagliflozin contribute to meaningful weight loss in overweight/obese T2DM patients?	254	44%	250	44%	30	5%	38	7%	572
7	Empagliflozin has evidence in non-diabetic heart failure and CKD patients. Do you believe it should be routinely considered?	234	41%	289	51%	12	2%	37	6%	572
8	Considering efficacy, safety, and real-world evidence, do you believe empagliflozin has a superior clinical profile to other SGLT2 inhibitors?	245	43%	229	40%	58	10%	40	7%	572
9	Given emerging SGLT2 inhibitor data in acute settings (e.g., heart failure, AKI prevention), should empagliflozin be used in hospitalized T2DM patients?	258	45%	211	37%	62	11%	41	7%	572
10	Do empagliflozin's clinical benefits outweigh concerns about rare adverse events like euglycemic DKA and genital infections in routine practice?	268	47%	224	39%	38	7%	42	7%	572
11	Do you believe empagliflozin should be used in T2DM patients without established ASCVD for primary cardiovascular disease prevention?	268	47%	219	38%	42	7%	43	8%	572
12	Do you agree that empagliflozin should be prioritized for reducing the risk of hospitalization for heart failure in T2DM patients with multiple risk factors?	205	36%	316	55%	4	1%	47	8%	572
13	Do you believe that empagliflozin's cardiovascular benefits also extend to T2DM patients with PAD, supporting its routine use?	284	50%	192	34%	48	8%	48	8%	572
14	Could empagliflozin, based on emerging evidence, be a therapy for MASLD and T2DM to reduce liver fat and fibrosis risk?	294	51%	199	35%	31	5%	48	8%	572
15	In your clinical experience, does empagliflozin significantly improve QoL in patients with T2DM and heart failure?	231	40%	288	50%	5	1%	48	8%	572
16	Would you consider empagliflozin in T2DM patients with hyperuricemia or a history of gout as an added benefit beyond glucose control?	305	53%	176	31%	41	7%	50	9%	572

For HF-related outcomes, 93% of respondents agreed that empagliflozin may be useful across the spectrum of ejection fraction. Similarly, 91% supported its use to help reduce HF-related hospitalizations in high-risk individuals, although a minority (1-11% across items) expressed disagreement or uncertainty. Renal protection also emerged as a prominent theme, with 93% agreeing that empagliflozin may help slow the progression of DKD. Early initiation in T2DM patients with high cardiorenal risk was supported by 90% of experts; however, 4% disagreed and 6% selected "NA," indicating that this perception was not universal.

More than 80% of clinicians acknowledged potential benefits related to blood pressure reduction, weight changes, metabolic dysfunction-associated steatotic liver disease (MASLD)-related parameters, and improvements in QoL, while a smaller proportion expressed reservations. Regarding safety, 86% of clinicians indicated that, in their experience, the benefits generally outweigh concerns related to rare adverse events such as genital infections or euglycemic diabetic ketoacidosis (DKA). Importantly, these responses represent clinician perceptions and practice impressions rather than comparative pharmacologic evidence. When asked to compare empagliflozin with other SGLT2 inhibitors, 83% perceived it as having a more favorable clinical profile based on their clinical practice experience; however, 10% disagreed and 7% responded "NA."

## Discussion

T2DM presents a growing global and national health burden, with a rising prevalence, particularly in countries like India. The disease is associated with significant morbidity and mortality, primarily due to its vascular complications, including ASCVD, HF, and DKD. In this context, SGLT2 inhibitors, especially empagliflozin, have gained prominence for their glycemic efficacy and pleiotropic benefits extending to cardiovascular and renal protection. The current consensus, derived from a nationwide expert advisory board, provides valuable real-world insights into the clinical positioning of empagliflozin across diverse patient profiles. The findings highlight a strong agreement among Indian clinicians on its foundational role in the management of T2DM, particularly in individuals with or at risk of cardiorenal complications. These perspectives align with global evidence and evolving treatment guidelines, reinforcing the need for early, targeted interventions in high-risk populations.

A strong consensus (95%) among surveyed clinicians endorsed empagliflozin as a foundational therapy for T2DM patients with established ASCVD which aligns with findings from the Empagliflozin Cardiovascular Outcome Event Trial in Type 2 Diabetes Mellitus Patients-Removing Excess Glucose (EMPA-REG OUTCOME) trial demonstrating a 38% reduction in cardiovascular deaths and a 35% reduction in HF hospitalization with empagliflozin [29,30]. A meta-analysis by Zelniker et al. [31] further confirmed empagliflozin's role in reducing major adverse cardiovascular events. Real-world Indian data have similarly reported favorable outcomes in glycemic control and cardiovascular risk reduction [32]. These findings reflect strong clinical confidence in the evidence base supporting empagliflozin's cardioprotective effects.

This expert opinion highlights clinical agreement (93%) on the use of empagliflozin for HF prevention in patients with T2DM, irrespective of ejection fraction status (HFrEF or heart failure with preserved ejection fraction (HFpEF)). This is consistent with clinical evidence from landmark trials such as the EMPA-REG OUTCOME, Empagliflozin Outcome Trial in Patients With Chronic Heart Failure and a Reduced Ejection Fraction (EMPEROR-Reduced), and Empagliflozin Outcome Trial in Patients With Chronic Heart Failure With Preserved Ejection Fraction (EMPEROR-Preserved), which demonstrated significant reductions in HF-related hospitalizations and cardiovascular death with empagliflozin in both reduced and preserved ejection fraction populations [16,30,33]. The early benefits observed suggest mechanisms beyond glycemic control, including natriuresis, reduced preload/afterload, and modulation of cardiac metabolism and inflammation. These findings strongly support integrating empagliflozin into standard care pathways for cardiometabolic risk reduction.

Empagliflozin has an increasingly important role in the multidisciplinary management of DKD, supported by randomized controlled trial (RCT) evidence demonstrating improvements in glycemic control along with clinically meaningful delays in DKD progression and reductions in end-stage renal disease and cardiovascular mortality [15,30]. Empagliflozin's renoprotective effects are attributed to reductions in intraglomerular pressure, increased natriuresis, and improved tubular workload, which collectively contribute to the observed delay in CKD progression and reduced incidence of renal endpoints [34]. The use of SGLT2 inhibitors is now recommended as part of treatment protocols based on international guidelines, including the Kidney Disease: Improving Global Outcomes (KDIGO) 2022, which advise their administration for patients with T2DM and CKD, regardless of glycemic control [26]. Evidences support the early use of SGLT2 inhibitors in T2DM patients with high cardiovascular or renal risk, and trials such as the EMPA-REG OUTCOME and Study of Heart and Kidney Protection With Empagliflozin (EMPA-KIDNEY) have shown that empagliflozin significantly reduces cardiovascular events and slows kidney disease progression, independent of glycemic control [30,35]. Reflecting this, the 2022 ADA/EASD consensus recommends SGLT2 inhibitors as first-line agents in high-risk patients, even before metformin [36]. Consistent with these recommendations, 90% of clinicians in the survey favored the early initiation of empagliflozin in such patients. In this opinion poll, 81% of clinicians recognized empagliflozin's blood pressure-lowering effect as a key factor in managing hypertensive T2DM patients which is supported by trials like the EMPA-REG OUTCOME, which demonstrated reductions in systolic blood pressure (BP) through osmotic diuresis and natriuresis, independent of glucose control [30,37]. These effects enhance its value in treating patients with both T2DM and hypertension.

A majority (92%) of clinicians endorsed the use of empagliflozin in non-diabetic patients with HF or CKD, reflecting its proven cardiorenal benefits beyond glycemic control. Trials like the EMPEROR-Reduced and EMPA-KIDNEY demonstrated significant reductions in HF hospitalizations and CKD progression, irrespective of diabetes status [15,33,38]. These findings support current guideline recommendations [26] and highlight empagliflozin's broader therapeutic role. Around 83% of clinicians favored empagliflozin over other SGLT2 inhibitors, citing its superior efficacy, safety, and real-world performance supported by the evidences from the EMPA-REG OUTCOME trial which demonstrated significant reductions in cardiovascular mortality and HF hospitalization [30], while real-world data from the Empagliflozin Comparative Effectiveness and Safety (EMPRISE) further supported its favorable profile [39]. Its high SGLT2 selectivity may also contribute to improved tolerability [40].

The use of empagliflozin in hospitalized patients with T2DM, particularly those presenting with acute HF or acute kidney injury (AKI), has garnered increasing attention due to emerging clinical trial data. In this poll, 82% of clinicians supported its use in such acute care settings, which is consistent with evidence from the Empagliflozin in Patients Hospitalized With Acute Heart Failure Who Have Been Stabilized (EMPULSE) trial that demonstrated significant clinical benefit when empagliflozin was initiated in patients hospitalized with acute HF, including improved clinical outcomes and QoL without an increase in adverse events [41]. Furthermore, analyses suggest SGLT2 inhibitors may offer renal protection even in acute settings, potentially mitigating AKI risk through hemodynamic stabilization and anti-inflammatory effects [42]. Despite 11% of respondents expressing caution, likely due to concerns about volume depletion or DKA, current data support the careful use of empagliflozin in selected hospitalized patients under appropriate clinical monitoring. Although concerns exist regarding rare adverse events such as euglycemic DKA and genital mycotic infections, the majority of clinicians (86%) in this opinion poll supported the favorable overall risk-benefit profile of empagliflozin. Evidence from the EMPA-REG OUTCOME trial and real-world studies further shows that these adverse effects can be minimized with appropriate patient selection and counseling [30,39]. Moreover, reductions in cardiovascular mortality, HF hospitalizations, and progression of renal disease observed with empagliflozin outweigh these risks in most patient populations [38,42]. This consensus affirms that, in routine clinical practice, the benefits of empagliflozin substantially surpass its safety concerns, reinforcing its role in comprehensive T2DM management.

HF is a major comorbidity in patients with T2DM, contributing significantly to morbidity and healthcare burden. Empagliflozin has demonstrated consistent benefits in reducing hospitalization for heart failure (HHF), as evidenced in the EMPA-REG OUTCOME trial, which showed a 35% relative risk reduction in HHF among T2DM patients with established cardiovascular disease [30]. Further trials, including the EMPEROR-Reduced and EMPEROR-Preserved, extended these benefits to patients across the spectrum of ejection fraction [16,33]. In line with this evidence, the present survey found that 91% of clinicians support prioritizing empagliflozin to reduce HF-related hospitalizations in high-risk T2DM patients. Peripheral artery disease (PAD) is a common macrovascular complication in patients with T2DM, significantly increasing the risk of cardiovascular morbidity and mortality. In this expert opinion, 84% of clinicians supported the routine use of empagliflozin in T2DM patients with PAD, reflecting confidence in its cardiovascular benefits within this high-risk subgroup. The EMPA-REG OUTCOME trial results showed a significantly reduced risk of major adverse cardiovascular events, including cardiovascular death, in T2DM patients with established ASCVD, which includes PAD [30]. Additionally, data suggest that SGLT2 inhibitors may improve vascular endothelial function, reduce arterial stiffness, and exert anti-inflammatory effects, which are relevant in PAD pathophysiology [43]. MASLD is a common comorbidity in T2DM, contributing to disease progression and increased cardiovascular risk. Empagliflozin has shown potential in reducing liver fat and fibrosis by improving insulin sensitivity and inducing weight loss [27,28,44]. In this expert opinion, 86% of clinicians recognized its emerging role in MASLD management. Supporting evidence from trials such as the Effect of Empagliflozin on Liver Fat Content in Patients With Type 2 Diabetes (E-LIFT) and real-world studies demonstrates significant improvements in hepatic steatosis and liver enzymes with empagliflozin therapy [45,46], highlighting its potential as a therapeutic option in this population.

Empagliflozin has demonstrated substantial benefits in improving QoL among patients with T2DM and HF. In this consensus, 90% of clinicians reported significant QoL improvements in such patients, consistent with findings from the trials, which showed not only reductions in HF hospitalizations but also meaningful enhancements in health status as measured by the Kansas City Cardiomyopathy Questionnaire (KCCQ) [16,33]. Additionally, 84% of experts supported the use of empagliflozin in T2DM patients with hyperuricemia or gout, attributing this to its uricosuric effects. Empagliflozin has been shown to reduce serum uric acid levels by increasing urinary excretion [47], which may help mitigate gout flares and in the long run contribute to cardiometabolic risk reduction.

A balanced interpretation of the expert responses also requires the careful consideration of empagliflozin's known safety profile. While most clinicians perceived the drug as having an acceptable safety profile, key adverse events such as genital mycotic infections, volume depletion, and the rare occurrence of euglycemic DKA remain clinically relevant. Appropriate patient selection, including avoiding use in individuals with significant dehydration, active ketosis, or prolonged fasting, is essential. Clinicians commonly recommend temporarily withholding therapy during acute illness, major surgery, or hemodynamic instability, which aligns with current sick-day rules and perioperative guidance. Ensuring adequate hydration, monitoring renal function, and counseling patients on genital hygiene further reduce risk. In hospitalized settings, early initiation may be considered only in clinically stable patients, with close monitoring of volume status and ketones. Incorporating these practical precautions provides a more balanced understanding of how clinicians weigh benefits against risks in real-world practice.

Despite the demonstrated benefits of empagliflozin in glycemic control, cardiovascular protection, and QoL, important evidence gaps remain. More long-term real-world data, particularly in diverse Indian populations, are needed to strengthen generalizability. Key limitations of this consensus include its reliance on expert opinion, potential response bias, and the absence of subgroup analyses by age, disease duration, or renal function. Additionally, the 16-item questionnaire used in this advisory board was not formally validated or pilot-tested, which may introduce measurement bias. The findings should therefore be interpreted as structured expert perceptions rather than definitive comparative evidence. In the Indian context, the applicability of these expert recommendations may also be influenced by real-world barriers such as medication cost, availability outside major urban centers, and variability in healthcare infrastructure, which can affect access and implementation. Future research should explore personalized treatment strategies, cost-effectiveness considerations, and the mechanisms underlying empagliflozin's pleiotropic effects.

## Conclusions

This consensus highlights strong clinical agreement among Indian experts regarding the multifaceted role of empagliflozin in managing T2DM, especially in patients with cardiovascular, renal, and metabolic comorbidities. Empagliflozin is not only recognized as a foundational therapy for patients with established ASCVD but also favored for early initiation in those at high cardiorenal risk. Its benefits extend beyond glycemic control, including improvements in HF outcomes, kidney protection, blood pressure reduction, weight management, QoL, and potential utility in conditions such as MASLD and hyperuricemia. Despite concerns around rare adverse events, clinicians consistently affirmed a favorable risk-benefit profile. These findings support the continued integration of empagliflozin into clinical practice and underscore the need for ongoing real-world evidence and research tailored to Indian patient populations.
